# Surgical Treatment of a Giant Popliteal Venous Aneurysm and Arteriovenous Fistula on the Adjacent Femoral Vein and Its Postoperative Findings

**DOI:** 10.3400/avd.cr.22-00044

**Published:** 2022-09-25

**Authors:** Atsushi Hiromoto, Shun-ichiro Sakamoto, Yusuke Motoji, Ryosuke Amitani, Takako Yamaguchi, Kenji Suzuki, Hiromasa Yamashita, Makoto Watanabe, Eitaro Kodani, Yosuke Ishii

**Affiliations:** 1 Department of Cardiovascular Surgery, Nippon Medical School Musashikosugi Hospital, Kawasaki, Kanagawa, Japan; 2 Department of Cardiovascular Surgery, Nippon Medical School Hospital, Tokyo, Japan; 3 Department of Cardiovascular Medicine, Nippon Medical School Tama Nagayama Hospital, Tokyo, Japan

**Keywords:** popliteal venous aneurysm, aneurysmorraphy, arteriovenous fistula

## Abstract

A case of a giant popliteal venous aneurysm that caused massive pulmonary thromboembolism with an arteriovenous fistula draining into the adjacent proximal femoral vein is reported herein. Deep veins in the lower leg were occluded by thrombi. The inlet and outlet orifice inside the aneurysm was closed and aneurysmorraphy was performed. The fistula was retained on the estimation that it would maintain the blood flow and prevent thrombus formation inside the femoral vein. The aneurysm was shrunk, the femoral vein was patent, and the fistula was not observed 1 year later, although it still existed 1 week after the operation.

## Introduction

Isolated venous aneurysms are defined as localized dilation communicating with the normal vein through a single channel.^1)^ Popliteal venous aneurysms (PVAs) frequently present with pulmonary thromboembolism (PTE), and symptomatic PVAs are managed surgically.^2–5)^ However, it is believed that PVA, accompanied by an arteriovenous fistula (AVF) draining into the adjacent femoral vein (FV) is rare, and its surgical treatment has not been reported. A surgical case of a giant PVA that presented with massive PTE, accompanied by a small AVF and its 1-year postoperative course, is reported herein.

## Case Report

**Figure 1:**
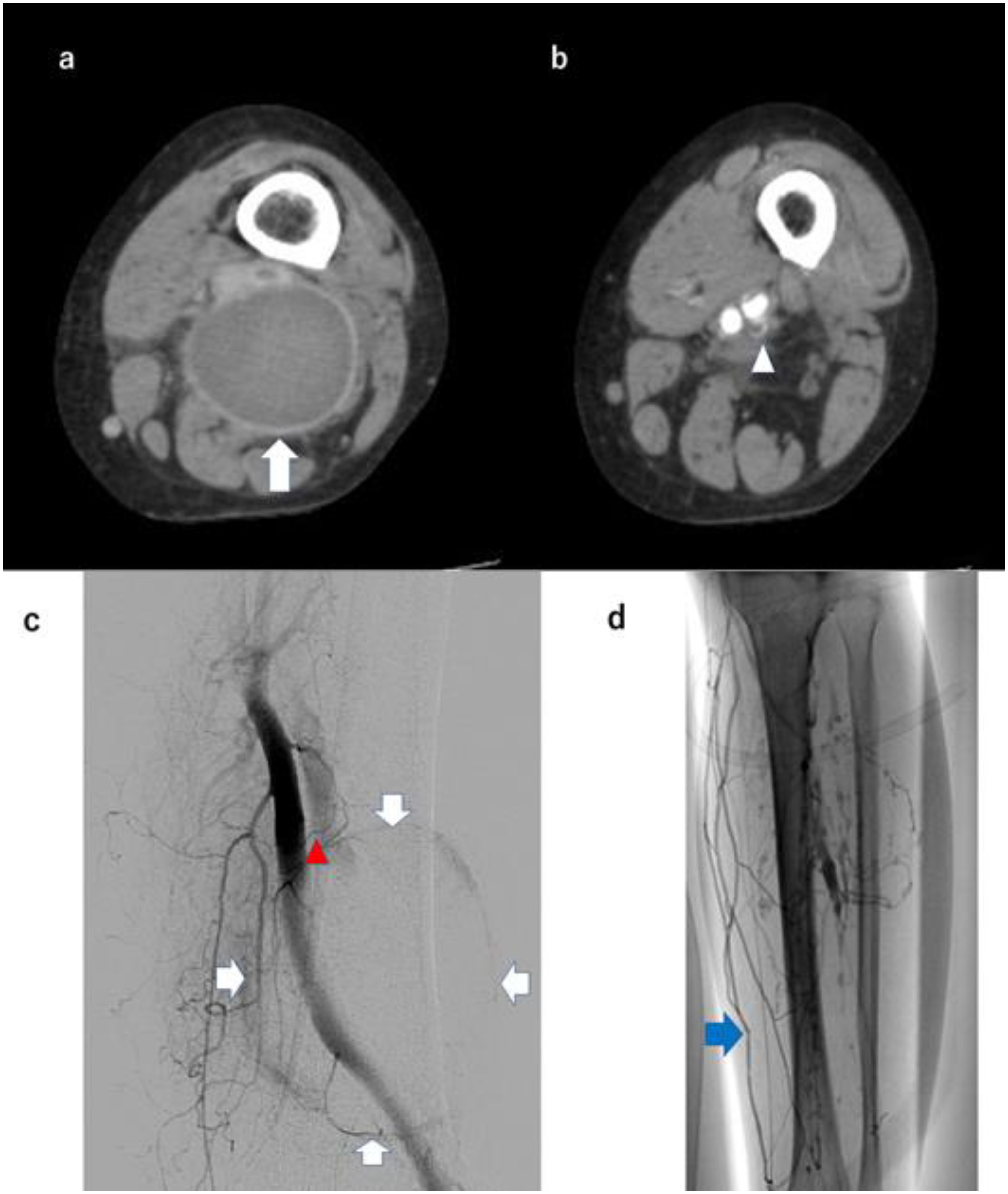
Fig. 1 (**a**) A giant fusiform PVA (white arrow) that is completely occluded by thrombi. (**b**) SFA and FV are simultaneously enhanced, indicating an AVF (white arrowhead). (**c**) In arteriography, SFA and FV are simultaneously enhanced via AVF (red arrowhead), and the surface of PVA is enhanced (white arrows). (**d**) In venography, a large part of the deep veins in the lower thigh is not enhanced. Venous return is dependent on the saphenous vein (blue arrow).

PVA: popliteal venous aneurysm; SFA: superficial femoral artery; FV: femoral vein; AVF arteriovenous fistula

A 61-year-old woman with a giant left PVA that had caused severe massive PTE necessitating extracorporeal membrane oxygenation was referred to the hospital of the current study. The patient’s chief complaint was a pain in the left lower thigh, probably due to compression of the surrounding tissue by the PVA. The maximum diameter of the PVA, filled with thrombi, was 53 mm on preoperative contrast-enhanced computed tomography (CT; **Fig. 1a**). A wide range of deep veins in the left lower leg and both the inlet and outlet veins of the PVA were filled with thrombi despite anticoagulation with rivaroxaban (**Fig. 1b**). In addition, a small AVF draining was observed from a branch of the superficial femoral artery (SFA) into the FV just adjacent to the PVA (**Fig. 1c**). Catheter arteriography and venography were conducted confirming AVF and showing that venous return of the lower thigh was dependent on the great saphenous vein (**Fig. 1d**).

**Figure 2:**
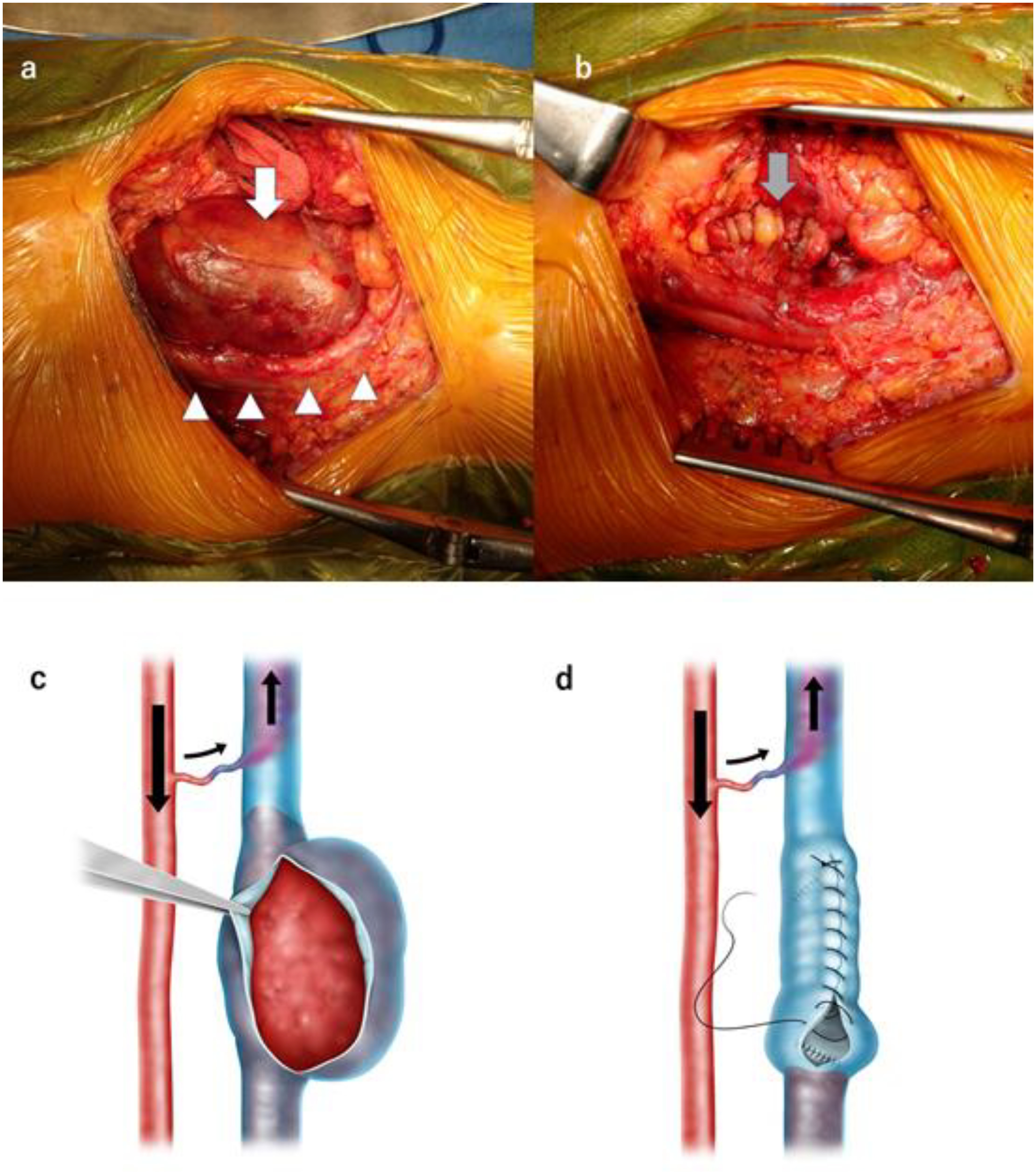
Fig. 2 (**a**) The PVA (white arrow) was exposed, and the sciatic nerve (white arrowheads) was gently retracted. (**b**) The redundant wall of the PVA was resected and the PVA was plicated (gray arrow). (**c**) The SFA and FV are connected by an AVF. (**d**) Both the inlet and the outlet of the PVA were closed and the aneurysm was plicated.

PVA: popliteal venous aneurysm; SFA: superficial femoral artery; FV: femoral vein; AVF arteriovenous fistula

These preoperative findings indicated that complete reconstruction of venous return in the deep vessels of the whole leg was impossible. Therefore, the indication for surgery, in this case, was to relieve the pain and prevent PTE recurrence. The PVA was approached via a sigmoid skin incision with the patient under general anesthesia and in the prone position. The sciatic nerve was attached to the PVA and then retracted gently and meticulously (**Fig. 2a**). The PVA was opened, and all the thrombi inside the aneurysm were removed. The aneurysmal wall was thick and had abundant vasa vasorum on its surface, implying an inflammatory response. Both the inlet and outlet inside the PVA were closed using running sutures. Identifying or closing the AVF was not attempted. The redundant aneurysmal wall was resected, and the aneurysm was plicated with running sutures (**Fig. 2b**). The scheme of the hemodynamics and the operative procedure is shown in **Figs. 2c** and **2d**.

**Figure 3:**
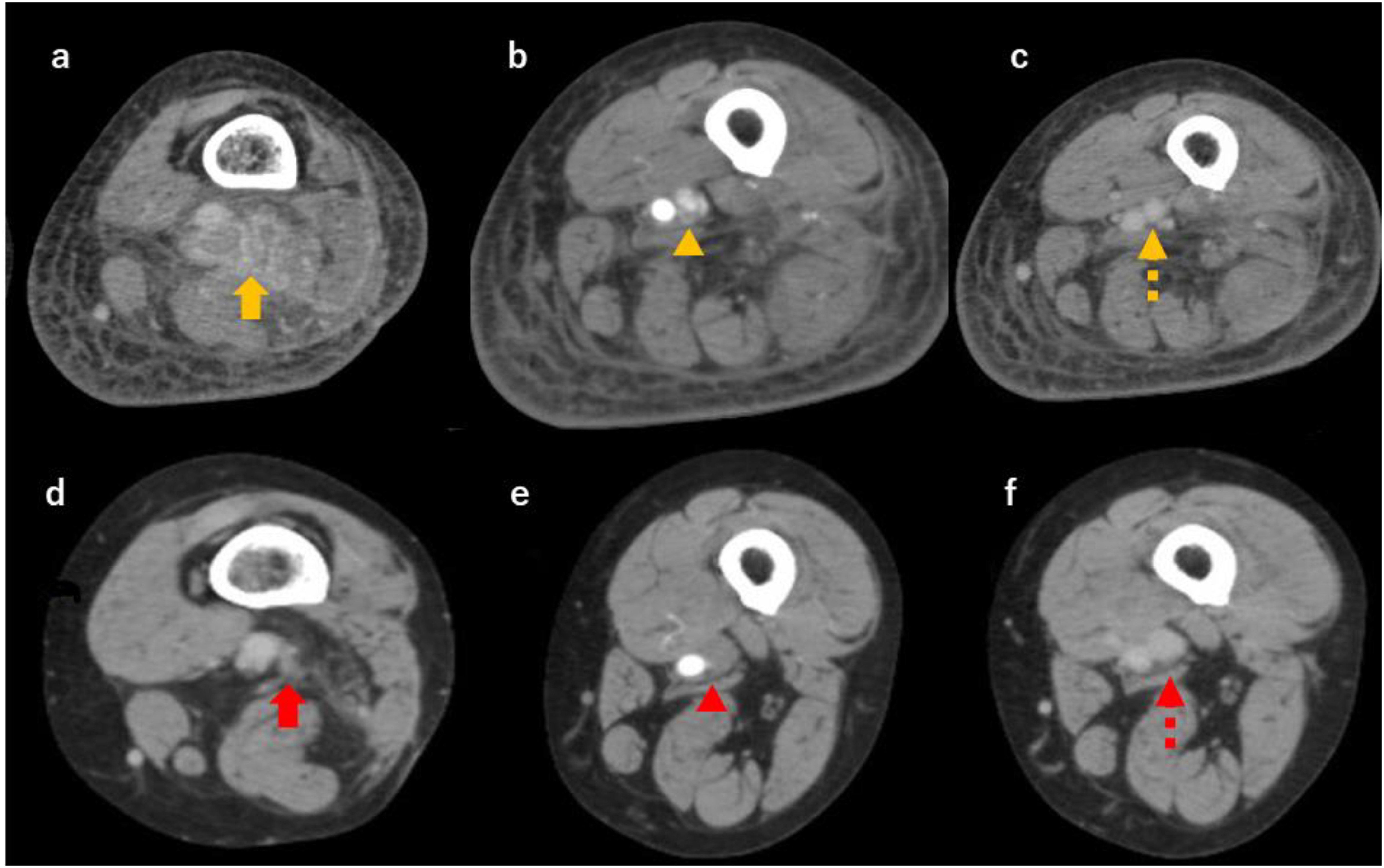
Fig. 3 (**a**) The plicated PVA is shown and its diameter is 15 mm (yellow arrow). (**b**) The AVF still exists (yellow arrowhead). (**c**) The FV is patent (yellow dotted arrow). (**d**) The plicated PVA is shrunk (red arrow). (**e**) The AVF is not shown (red arrowhead). (**f**) The FV is patent (red dotted arrow).

PVA: popliteal venous aneurysm; AVF arteriovenous fistula; FV: femoral vein

The diameter of the plicated aneurysm was 15 mm (**Fig. 3a**), and the fistula was preserved (**Fig. 3b**) 1 week after the operation as shown in CT. The FV was enhanced via the AVF and was patent (**Fig. 3c**). The patient was discharged without any complications and was free from pain.

The patient underwent CT again 1 year after the operation. The plicated PVA was shrunk and seemed almost like a scar (**Fig. 3d**). Interestingly, the fistula was not observed (**Fig. 3e**). The patency of the whole FV was maintained (**Fig. 3f**).

## Discussion

PVAs are uncommon but potentially cause pain, swelling, edema, and severe PTE. Surgery is indicated in all symptomatic PVA cases because of the high risk of PTE.^4)^ As a preoperative diagnostic modality, a duplex ultrasound scan is reported to be the first choice,^2)^ and magnetic resonance imaging and venography are also utilized. Gabrielli et al. stated that CT is mandatory for investigating the deep venous system and defining venous anatomy.^1)^ In the current case, preoperative CT revealed a small AVF draining from a branch of the SFA to the FV adjacent to the PVA. Angiography confirmed the existence of the AVF and venous return depending on the great saphenous vein and deep veins in the thigh. The PVA and a large part of the deep veins in the lower leg were filled with thrombi.

In this case, two indications for the surgery were observed. The first was preventing PTE recurrence, and the second was pain relief. Several surgical approaches for treating PVA have been reported. They consist of tangential aneurysmectomy and lateral venorraphy (TALV), resection with autologous vein graft interposition, resection and end-to-end anastomosis, and simple ligation or resection.^2–5)^ Emmerich reported the efficacy of FV ligation just below the confluence of the great saphenous vein and the deep FV.^6)^ For PTE prevention, the aneurysm was closed by suturing both the inlet and the outlet of the aneurysm. An AVF is sometimes created concomitantly to achieve and maintain venous patency by creating an arterial communication to drive venous flow when thrombectomy of a deep vein is performed.^7,8)^ The preservation of the AVF was surmised to contribute to maintaining FV patency by a similar mechanism. Therefore, this anomaly was intentionally preserved and the FV was not ligated. For pain relief, TALV was performed and aneurysm volume was reduced. Consequently, FV patency was maintained and the aneurysm was well shrunk in the CT 1 year after the operation. Currently, the patient is free from pain. Interestingly, the AVF was not observed in CT 1 year after the operation although it still existed 1 week after the operation.

How the small AVF was formed in this case is still unclear. Local inflammation has been reported to be a mechanism of AVF formation.^9)^ As stated above, the finding of the aneurysmal wall indicated an inflammatory response. In this case, it is estimated that inflammation around the aneurysm caused AVF formation, and the AVF disappeared after healing of the inflammation.

The blood flow inside the AVF was not measured, and its quantitative change is unknown. However, a comparison between **Figs. 1b** and **3b** suggests that the blood flow ratio between the AVF and the FV changed because FV enhancement via the AVF was weaker postoperatively than it was preoperatively. Edema of the leg was reduced in the long term postoperatively, and it is inferred that venous return gradually improved and that AVF disappeared after its blood flow fell below the FV flow. The preservation of the AVF is believed to somehow be attributed to preventing thrombus formation, at least in the postoperative early phase when the AVF flow was still higher than the FV flow.

It is believed that the coexistence of a giant PVA and an adjacent AVF has not been clearly reported. From this point of view, this report has its significance in manifesting the postoperative course of such a case by findings in CT.

The strategy of postoperative anticoagulation has not yet been established in the literature.^2–5)^ In this case, anticoagulation with rivaroxaban is continued to prevent thrombus formation inside the FV. AVF disappearance has been observed, and the continuation of oral anticoagulation is believed to be validated. Further follow-up is required to ascertain the long-term FV patency.

## Conclusion

In surgical PVA treatment, the hemodynamics and anatomy should be thoroughly evaluated at the preoperative stage. CT or angiography should be performed because they may provide essential information. In such a case as the coexistence of a giant PVA and an adjacent AVF, complete closure of the aneurysm with TALV, and postoperative anticoagulation provided satisfactory results even though the fistula was untouched.

## List of Abbreviations

PVA: popliteal venous aneurysm
PTE: pulmonary thromboembolism
AVF: arteriovenous fistula
FV: femoral vein
CT: computed tomography
SFA: superficial femoral artery
TALV: tangential aneurysmectomy and lateral venorraphy
